# Experimental observation of spatially resolved photo-luminescence intensity distribution in dual mode upconverting nanorod bundles

**DOI:** 10.1038/srep42515

**Published:** 2017-02-13

**Authors:** Pawan Kumar, Satbir Singh, V. N. Singh, Nidhi Singh, R. K. Gupta, Bipin Kumar Gupta

**Affiliations:** 1 Luminescent Materials and Devices Group, Materials Physics and Engineering Division, CSIR- National Physical Laboratory, Dr. K. S. Krishnan Road, New Delhi, 110012, India; 2 Academy of Scientific and Innovative Research (AcSIR), CSIR-National Physical Laboratory Campus. Dr. K. S. Krishnan Road, New Delhi 110012, India; 3 Advanced Materials and Devices Group, Physics of Energy Harvesting Division, CSIR - National Physical Laboratory, Dr. K. S. Krishnan Road, New Delhi, 110012, India; 4 Metals, Alloys and Composites for Energy Applications Group, Physics of Energy Harvesting Division, CSIR - National Physical Laboratory, Dr K S Krishnan Road, New Delhi, 110012, India; 5 Department of Chemistry, Pittsburg State University, Pittsburg, KS, 66762, USA

## Abstract

A novel method for demonstration of photoluminescence intensity distribution in upconverting nanorod bundles using confocal microscopy is reported. Herein, a strategy for the synthesis of highly luminescent dual mode upconverting/downshift Y_1.94_O_3_:Ho^3+^_0.02_/Yb^3+^_0.04_ nanorod bundles by a facile hydrothermal route has been introduced. These luminescent nanorod bundles exhibit strong green emission at 549 nm upon excitations at 449 nm and 980 nm with quantum efficiencies of ~6.3% and ~1.1%, respectively. The TEM/HRTEM results confirm that these bundles are composed of several individual nanorods with diameter of ~100 nm and length in the range of 1–3 μm. Furthermore, two dimensional spatially resolved photoluminescence intensity distribution study has been carried out using confocal photoluminescence microscope throughout the nanorod bundles. This study provides a new direction for the potential use of such emerging dual mode nanorod bundles as photon sources for next generation flat panel optical display devices, bio-medical applications, luminescent security ink and enhanced energy harvesting in photovoltaic applications.

Designing one-dimensional (1D) rare earth nanomaterials by template-free strategies is an ultimate challenge of cutting edge science^1^,^2^,^3^,^4^,^5^,^6^. In general, the chemical, physical and optical properties of inorganic nanostructures depend on their chemical composition, size and shape^6^,^7^,^8^,^9^,^10^,^11^,^12^. In recent times, rare earth-doped nanostructures have been recognized worldwide for their better chemical and optical properties originating from their unique electronic structures as well as wide range of applications in photovoltaic, bio-medical, anti-counterfeiting, solid state lighting, display technologies etc^13^,^14^,^15^,^16^. In comparison to organic dyes, metals, metal oxides, semiconductor quantum dots and core-shell structures; rare earth compounds present intense and sharp emission bands arising from f–f transitions and large Stokes shifts originating from their unique electronic configuration^17^,^18^,^19^,^20^,^21^,^22^. The 1D nanostructures of rare earth doped nanomaterials (like nanorods, nanowires, nanotubes etc.) have attracted enormous attention in recent years^23^,^24^,^25^,^26^. Tailoring of aspect ratio in rare earth based 1D nanostructure offers several advantages; like, quantum confinement, tunable electrical, magnetic and optical properties^27^. There are many reports on the synthesis of 1D nanomaterials; such as, III-V and II-VI semiconductors and oxide nanowires/nanorods^28^,^29^,^30^,^31^. The widely used methods to prepare 1D structures are catalyst supported template as well as chemical vapour deposition. But, these methods have their own drawbacks; such as, complex procedure and impurities in the products. Therefore, the solution-phase methods for direct growth of 1D nanostructure without involving catalysts or templates (such as hydrothermal method) are widely used for the synthesis of 1D nanomaterials with better purity, large scale production at economical cost and good homogeneity^15^. Now a days, bundle composed of rare earth based nanorods have gained much attention due to higher surface area, better quantum yield and optical properties^1^,^13^,^15^,^32^. These bundles of nanorods could be synthesized by using customized hydrothermal method without any external assistance^32^. Moreover, these luminescent bundles are highly desired for fabrication of flat panel optical display devices, which ignited us to explore the synthesis as well as spatially resolved photoluminescence (PL) intensity distribution on the surface of these nanorod bundles.

Recently, the upconversion nanomaterials have received huge attention due to their various potential applications^33^,^34^. It is well established that the upconversion process involves an anti-Stokes shift in which absorption of multi-photons (two or more) of lower energy (infrared photons) results into emission of high energy photons. The upconverting phosphors are generally inorganic host lattice (chosen due to their low phonon energy) doped with emitters Er^3+^, Tm^3+^, Ho^3+^ and Yb^3+^ 34,35,36. Y_2_O_3_ is a one of the most explored host lattice due to its exceptional optical, thermal and mechanical properties^37^,^38^,^39^. In addition to this, the nucleation and formation of hexa-hydroxy rods and their conversion into oxide nanorods is quite easy in the binary system as compared to ternary system e.g. GdVO_4_, NaYF_4_, LaPO_4_ etc. Various synthesis methods have been used for the growth of upconverting nanophosphor with different morphologies; like, nanoparticles, nanotubes, nanoflakes, nanorods etc^40^,^41^,^42^. Moreover, it is interesting to note that the bundles composed of rare earth based nanorods with dual mode emission (both downshift/down conversion as well as upconversion) are meagrely reported in literature. These dual mode nanorod bundles open a new paradigm shift from nanorod to nanorod bundle structure for highly efficient next generation optical display applications. In order to establish a potential use of such luminescent nanorod bundles, it is extremely important to investigate the PL intensity distribution throughout the surface of nanorod bundles. Confocal PL mapping microscopy has gained recognition for the visualization of 2D spatial distribution of PL intensity in luminescent materails^43^,^44^. Furthermore, PL mapping provides a mapped image by integrating thousands of acquired PL spectra at every point and gives information about spectroscopic features at that particular point. Conceptually, image formation by PL mapping involves measuring a property from the entire field of view concurrently or by measuring a property of entire area sequentially from each points and combining it to recreate the image^45^. Hence, PL mapping is an important tool to explore the PL intensity distribution throughout the surface of nanorod bundles. There are few reports describing the charge distribution in organic field effect transistor, spatially resolved doping, non-radiative lifetime profiles in single Si-doped InP nanowires etc^43^,^44^. However, studies on spatially resolved PL intensity distribution in the rare earth based luminescent nanorod bundles or any other morphology related to rare earths are still not focused. While, most of the display devices are based on rare earth based luminescent materials; e.g. YAG:Ce coated on blue LEDs for white light generation and similarly many other displays. The measurements of spatial PL intensity distribution on phosphor coated surfaces and its standardization can easily be observed through confocal PL mapping. The uniformity of PL intensity distribution of luminescent materials coated surface is an important parameter for deciding the performance of optical devices. Therefore, the PL intensity distribution study (using photoluminescence confocal microscopy) can bring a better understanding about the spatial PL intensity distribution in luminescent materials (beyond the naked eye limit) and provides a new direction that is extremely important for next generation photo emission based displays applications.

In this article, synthesis of dual mode Y_1.94_O_3_:Ho^3+^_0.02_/Yb^3+^_0.04_ nanorod bundles by a facile hydrothermal method has been reported. The Y_1.94_O_3_:Ho^3+^_0.02_/Yb^3+^_0.04_ nanorod bundles emit strong green colour centred at 549 nm upon excitations with of 449 nm and 980 nm wavelengths. The structural analysis of these nanorod bundles have been carried out using X-ray diffraction (XRD). The morphological and microstructural investigations of these luminescent nanorod bundles have been performed by scanning electron microscopy (SEM) and transmission electron microscopy (TEM)/high-resolution transmission electron microscopy (HRTEM) techniques. Further, 2D spectral distribution of PL intensity of these luminescent nanorod bundles have been investigated for the first time.

## Results

**Figure 1:**
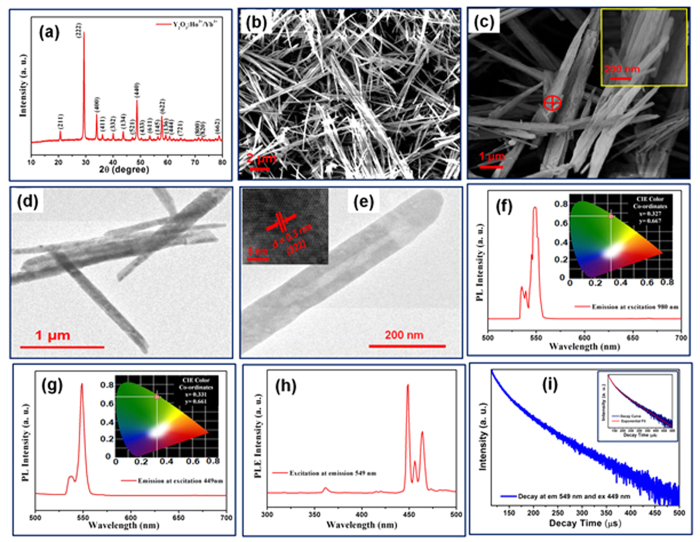
(**a**) XRD pattern of Y_1.94_O_3_: Ho^3+^_0.02_/Yb^3+^_0.04_ nanorod bundles. (**b**) SEM image of Y_1.94_O_3_:Ho^3+^_0.02_/Yb^3+^_0.04_ nanorod bundles. (**c**) The magnified view of SEM image nanorod bundles and inset exhibits the further magnified view of red marked region in (**c**). (**d**) TEM image of nanorod bundle taken from selected area. (**e**) TEM micrograph of individual nanorod and inset shows the HRTEM of nanorod. (**f**) PL emission spectrum of Y_1.94_O_3_:Ho^3+^_0.02_/Yb^3+^_0.04_ nanorod bundles at excitation wavelength of 980 nm and inset demonstrates CIE colour coordinates for green emission. (**g**) PL emission spectrum of Y_1.94_O_3_:Ho^3+^_0.02_/Yb^3+^_0.04_ nanorod bundles at excitation wavelength of 449 nm and inset demonstrates CIE colour coordinates of green emission. (**h**) PL excitation spectrum of Y_1.94_O_3_:Ho^3+^_0.02_/Yb^3+^_0.04_ nanorod bundles at emission wavelength of 549 nm. (**i**) TRPL decay profile of nanorod bundles recorded at room temperature while monitoring emission at 549 nm, at an excitation of 449 nm and inset shows the exponential fitting of the decay profile.

Y_1.94_O_3_:Ho^3+^_0.02_/Yb^3+^_0.04_ nanorod bundles were synthesized by a facile hydrothermal method. Details of the synthesis process for Y_1.94_O_3_:Ho^3+^_0.02_/Yb^3+^_0.04_ nanorod bundles have been described in experimental section. The structural analysis of Y_1.94_O_3_:Ho^3+^_0.02_/Yb^3+^_0.04_ nanorod bundles (before and after sintering) was investigated using X-ray diffraction (XRD) technique. The XRD pattern of Y_1.94_(OH)_3_: Ho^3+^_0.02_/Yb^3+^_0.04_ nanorod bundles is illustrated in Figure S1 (see Supplementary Information). The XRD results reveal that Y_1.94_(OH)_3_:Ho^3+^_0.02_/Yb^3+^_0.04_ nanorod bundles have hexagonal structure with space group P6_3_/m (JCPDS card no. 83–2042). The lattice parameters of nanorod bundles were calculated from observed d-values using a least-squares fitting method (using unit cell refinement software)^46^. The calculated lattice parameters for Y_1.94_(OH)_3_:Ho^3+^_0.02_/Yb^3+^_0.04_ nanorod bundles are, a = b = 6.2384 ± 0.0040 Å and c = 3.5276 ± 0.0043 Å with cell volume of 118.8947 ± 0.0183 Å^3^, which is comparable to the standard lattice parameters, a = b = 6.2610 Å, c = 3.5440 Å & cell volume of 120.3100 Å^3^(JCPDS card no. 83–2042). Figure 1a demonstrates the XRD pattern of Y_1.94_O_3_:Ho^3+^_0.02_/Yb^3+^_0.04_ nanorod bundles sintered at 1000 °C. The XRD results reveal that Y_1.94_O_3_:Ho^3+^_0.02_/Yb^3+^_0.04_ nanorod bundles have cubic structure (JCPDS card no. 43–1036). The estimated lattice parameters of Y_1.94_O_3_:Ho^3+^_0.02_/Yb^3+^_0.04_ nanorod bundles are, a = b = c = 10.5841 ± 0.0125 Å and cell volume is 1185.6720 ± 4.2106 Å^3^ which matches well with standard lattice parameters of a = 10.6040 Å and cell volume of 1192.36 Å^3^. Figure S2 (see Supplementary Information) shows proposed cubic crystal structure of Y_1.94_O_3_:Ho^3+^_0.02_/Yb^3+^_0.04_ nanorod bundles, where Y atoms are substituted by Ho and Yb atoms in unit cell as per coordination number and ratio of Ho/Yb. Further, crystal structure and phase of Y_1.94_O_3_:Ho^3+^_0.02_/Yb^3+^_0.04_ nanorod bundles were investigated using Raman spectroscopy. Figure S3 (see Supplementary Information) shows the Raman spectrum of Y_1.94_O_3_:Ho^3+^_0.02_/Yb^3+^_0.04_ nanorod bundles exhibiting peaks at 128, 161, 193, 230, 293, 304, 332, 378, 433, 470 and 593 cm^−1^. The intense peak at 378 cm^−1^ represents cubic structure of Y_1.94_O_3_:Ho^3+^_0.02_/Yb^3+^_0.04_ nanorod bundles^47^,^48^. Furthermore, thermogravimetric analysis (TGA) was performed to examine the thermal decomposition of Y_1.94_O_3_:Ho^3+^_0.02_/Yb^3+^_0.04_ nanorod bundles. Figure S4 (see Supplementary Information) shows the TGA graph of as prepared Y_1.94_(OH)_3_:Ho^3+^_0.02_/Yb^3+^_0.04_ nanorod bundles. TGA graph demonstrates the total weight loss during heating is 25.99%. Moreover, TGA graph exhibits the weight loss occurred in three steps upto 900 °C. The weight loss in the first step was 2.91% which is attributed to the transformation of polymer complexed metal nitrate conversion into metal hydroxide (upto~252 °C). In the second step, a major weight loss upto 13.65% was observed which is related to the dehydration of hydroxide and formation of oxide nanorod bundles (upto~363 °C). In the final step, till 800 °C, weight loss of 9.43% was observed which is ascribed to removal of unused intercalated nitrates ions^32^.

The scanning electron microscopy (SEM) was used to probe the surface morphology of nanorod bundles. SEM image of Y_1.94_(OH)_3_: Ho^3+^_0.02_/Yb^3+^_0.04_ nanorod bundles is shown in Figure S5 (see Supplementary Information). Figure 1b demonstrates SEM image of Y_1.94_O_3_:Ho^3+^_0.02_/Yb^3+^_0.04_ nanorod bundles. The SEM image clearly shows uniform growth of nanorod bundles throughout the sample with diameter in the range of 0.4 to 0.6 μm and length from 6 to 10 μm. The magnified SEM image of nanorod bundles is shown in Fig. 1c, which clearly demonstrates high density of nanorods inside bundles. The inset of Fig. 1c exhibits that the diameter of individual nanorod is ~100 nm (estimated using red marked portion in Fig. 1c). Inset shows that the diameter of nanorod is ~100 nm. Further, the elemental analysis of Y_1.94_O_3_:Ho^3+^_0.02_/Yb^3+^_0.04_ nanorod bundles was investigated using energy dispersive X-ray analysis (EDAX). The EDAX spectrum was taken from the red mark region in Fig. 1c. The EDAX spectrum of Y_1.94_O_3_:Ho^3+^_0.02_/Yb^3+^_0.04_ nanorod bundles is shown in Figure S6 (see Supplementary Information). The EDAX spectrum of nanorod bundles confirms the presence of Y, O, Yb and Ho elements. In order to explore the microstructural information of bundles as well as individual nanorod, the transmission electron microscope (TEM) was used. Figures 1d and S7 (see Supplementary Information) exhibit TEM image of nanorod bundles from different areas. Furthermore, in order to explore the dimensions of individual nanorod, the powder sample of Y_1.94_O_3_:Ho^3+^_0.02_/Yb^3+^_0.04_ nanorod bundles was dispersed in ethanol and ultra-sonicated at 45 kHz for 45 min, prior to TEM characterization to avoid any agglomeration between nanorods. Figure 1e demonstrates TEM micrograph of individual nanorod.TEM image of nanorod reveals that these nanorods have diameter of ~100 nm. The HRTEM image of nanorod is shown in inset of Fig. 1e, which exhibits that the nanorod has well resolved fringes without lattice distortion. The estimated d-spacing of nanorod is ~0.3 nm which is comparable to the value of 0.306 nm corresponding to (222) plane of Y_2_O_3_ (JCPDS card no. 43–1036). A plausible mechanism for the growth of nanorod bundles is demonstrated in Figure S8 (see Supplementary Information). The nucleation mechanism of nanorod bundles depends upon three major parameters; pH of metal precursor solution, growth temperature and capping agent. The proposed mechanism for nanorod bundles growth involves two major steps: formation of nucleation center during metal nitrates complex formation with CTAB at higher pH ~13 and formation of hexa-hydroxide nanorod bundles^15^ at 185 °C during hydrothermal process for 8 hours. Higher pH (~13) of metal nitrates precursor solution facilitate growth of rod like morphology due to rapid formation of anisotropic aggregate nucleation centers which initiates growth of several co-nuclei centers. The rapid growth conditions along with co-nuclei effect favours the formation of an-isotropic structure inthe bundle form^15^. Finally, the hydroxide nanorod bundles get converted into oxide nanorod bundles during sintering at high temperature of ~1000 °C for 5 hours.

The spectroscopic features of Y_1.94_O_3_:Ho^3+^_0.02_/Yb^3+^_0.04_ nanorod bundles were examined using PL and TRPL (time-resolved photoluminescence) spectroscopic techniques. Figure 1f exhibits the emission spectrum of Y_1.94_O_3_:Ho^3+^_0.02_/Yb^3+^_0.04_ nanorod bundles upon excitation with 980 nm wavelength. The PL emission spectrum of Y_1.94_O_3_:Ho^3+^_0.02_/Yb^3+^_0.04_ nanorod shows strong green emission peaking at 549 nm which corresponds to ^5^F_4_, ^5^S_2_-^5^I_8_ transition in Ho^3+^ ion^49^,^50^. The green emission of Y_1.94_O_3_:Ho^3+^_0.02_/Yb^3+^_0.04_ nanorod bundles can be explained using two processes; energy transfer upconversion (ETU) and excited-state absorption (ESA). In the energy transfer upconversion (ETU) process, the sensitizer ion (Yb^3+^) and activator ion (Ho^3+^)absorb the laser photons (pump photon) and get excited to higher energy state (metastable state). The excited sensitizer ion (Yb^3+^) transfer its energy to activator ion (Ho^3+^) at meta-stable state and relax back to ground state. This transferred energy excite meta-stable activator ion (Ho^3+^) to higher energy level. In excited state absorption (ESA) process, activator ions absorb two photons. The first photon excites the activator ion to meta-stable state and the second photon promotes it to higher excited state, which is responsible for upconversion. Figure S9 (see Supplementary Information) demonstrates the proposed energy level diagram for upconversion inY_1.94_O_3_:Ho^3+^_0.02_/Yb^3+^_0.04_ nanorod bundles. The inset in Fig. 1f demonstrates the CIE color co-ordinates corresponding to emission spectrum of Y_1.94_O_3_:Ho^3+^_0.02_/Yb^3+^_0.04_ nanorod bundles under excitation at 980 nm wavelength, where x = 0.327 and y = 0.667. Figure 1g exhibits the downconversion/downshift PL emission spectrum of Y_1.94_O_3_:Ho^3+^_0.02_/Yb^3+^_0.04_ nanorod bundles at excitation of 449 nm. The PL emission spectrum of nanorod bundles shows strong green emission at 549 nm. The inset in Fig. 1g demonstrates the CIE color co-ordinates of emission spectrum at excitation of 449 nm, where x = 0.331 and y = 0.661. The CIE coordinates of both downshift and upconversion of Y_1.94_O_3_:Ho^3+^_0.02_/Yb^3+^_0.04_ nanorod bundles are almost equal at 549 nm. The excitation spectrum of nanorod bundles at fixed emission wavelength of 549 nm is illustrated in Fig. 1h. The proposed energy level diagram for downshift in Y_1.94_O_3_:Ho^3+^_0.02_/Yb^3+^_0.04_ nanorod bundles is demonstrated in Figure S10 (see Supplementary Information). Further, the quantum efficiency of dual mode Y_1.94_O_3_:Ho^3+^_0.02_/Yb^3+^_0.04_ nanorod bundles for downshift and upconversion are ~6.3% (using xenon lamp as source of excitation) and ~1.1% (using 980 nm diode laser with power density 150 Wcm^−2^), respectively.

Further, the time-resolved photoluminescence (TRPL) was recorded using a single photon counting technique using a microsecond xenon flash lamp as the source of excitation. The application of luminescent materials depends upon the observed lifetime. It is well established that lifetime in the range of milliseconds to microseconds are highly useful for several potential applications; such as optical display devices, bio-medical and security ink applications^1^,^14^,^15^,^16^,^46^. The semi-logarithmic (logarithmic scale on the y-axis and linear scale on the x-axis) decay profile of Y_1.94_O_3_:Ho^3+^_0.02_/Yb^3+^_0.04_ nanorod bundles at emission wavelength of 549 nm upon excitation wavelength of 449 nm is demonstrated in Fig. 1i. The decay profile of Y_1.94_O_3_:Ho^3+^_0.02_/Yb^3+^_0.04_ nanorod bundles is best fitted with double exponential function as given in equation (1)^51^,^52^. The inset of Fig. 1i shows the fitted exponential curve of decay profile. The parameters generated form exponential fitting of decay profile are τ_1_ = 20.84 μs, τ_2_ = 80.45 μs, χ2 = 1.44, A_1_ = 35 and A_2_ = 65. The decay time of nanorod bundles are τ_1_ = 20.84 μs and τ_2_ = 80.45 μs.The double exponential decay components of Y_1.94_O_3_:Ho^3+^_0.02_/Yb^3+^_0.04_ nanorod bundles indicating the presence of at least two electronically excited species. The presence of activator Ho^3+^ ion and co-activator/sensitizer Yb^3+^ ion in Y_2_O_3_ host lattice could be the reason behind the observed double exponential decay. The average decay time of nanorod bundles is τ_av_ = 61.33 μs, which is calculated using equation (2)^50^,^51^.


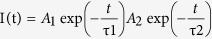



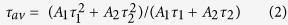


The obtained spectroscopic results reveal that the Y_1.94_O_3_:Ho^3+^_0.02_/Yb^3+^_0.04_ nanorod bundles have dual mode (downshift as well as upconversion) upon excitation wavelengths of 980 nm and 449 nm. The observed lifetime results suggest that these nanorod bundles are highly useful for various applications such as advanced optical display devices^16^, bio-medical^1^,^14^ and security ink applications^1^,^15^,^46^.

**Figure 2:**
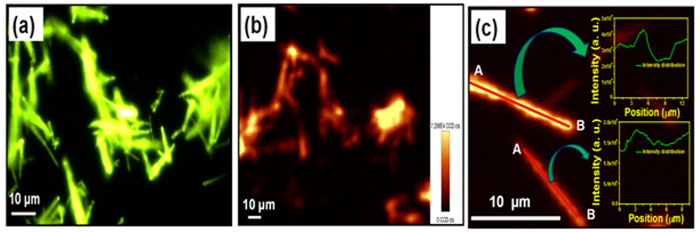
(**a**) Fluorescent image of Y_1.94_O_3_:Ho^3+^_0.02_/Yb^3+^_0.04_ nanorod bundles at excitation wavelength of 980 nm. (**b**) PL mapping image of nanorod bundles at excitation wavelength of 980 nm at same location from where the fluorescent image was taken. (**c**) PL mapping image of two nanorod bundles at two different places with different profile heights, insets show the PL intensity distribution at different positions.

**Figure 3:**
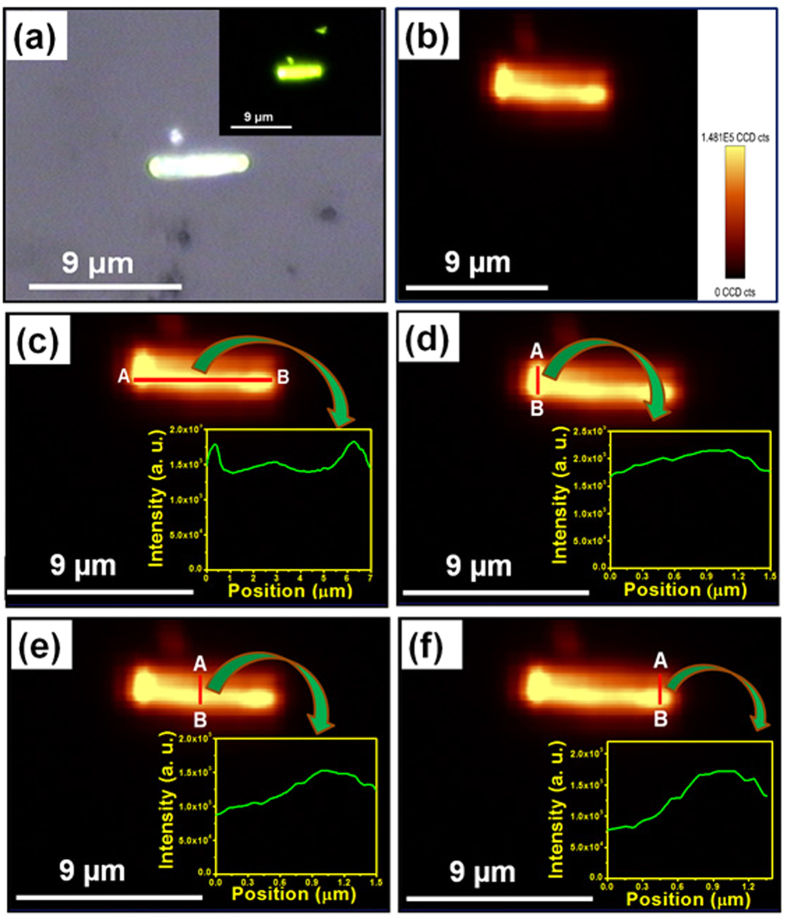
(**a**) Optical image of isolated horizontal positioned single Y_1.94_O_3_:Ho^3+^_0.02_/Yb^3+^_0.04_ nanorod bundle and inset shows fluorescent image of isolated horizontally positioned single Y_1.94_O_3_:Ho^3+^_0.02_/Yb^3+^_0.04_ nanorod bundle at excitation wavelength of 980 nm. (**b**) PL mapping image of isolated horizontally positioned single Y_1.94_O_3_:Ho^3+^_0.02_/Yb^3+^_0.04_ nanorod bundle. (**c**) PL intensity distribution along the length of nanorod bundle from one end to other end of nanorod bundle (A to B). (**d–f**) PL intensity distribution along the diameter of isolated bundle at three different selected positions; left, middle and right sides (A to B), respectively.

**Figure 4:**
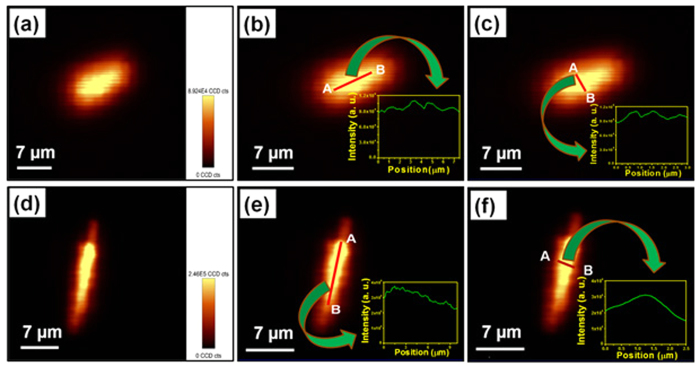
(**a**) PL mapping of slanted positioned Y_1.94_O_3_:Ho^3+^_0.02_/Yb^3+^_0.04_ nanorod bundle. (**b**) PL mapping along the length of slant positioned Y_1.94_O_3_:Ho^3+^_0.02_/Yb^3+^_0.04_ nanorod bundle, inset shows the intensity distribution from position A to B. (**c**) PL mapping along the diameter of slant positioned Y_1.94_O_3_:Ho^3+^_0.02_/Yb^3+^_0.04_ nanorod bundle, inset shows the intensity distribution from position A to B. (**d**) PL mapping vertically positioned Y_1.94_O_3_:Ho^3+^_0.02_/Yb^3+^_0.04_ nanorod bundle. (**e**) PL mapping along the length of vertically positioned Y_1.94_O_3_:Ho^3+^_0.02_/Yb^3+^_0.04_ nanorod bundle, inset shows the intensity distribution from position A to B. (**f**) PL mapping along the diameter of vertically positioned Y_1.94_O_3_:Ho^3+^_0.02_/Yb^3+^_0.04_ nanorod bundle, inset shows the intensity distribution from position A to B.

The 2D spatially resolved PL mapping was performed to explore the PL intensity distribution in nanorod bundles. The use of confocal microscope for PL imaging allowed mapping the spatial variation in the PL intensity of nanorod bundles using an excitation wavelength of 980 nm. The schematic diagram and theoretical concept of confocal microscope is shown in Figure S11 (see Supplementary Information). Figure 2a represents the fluorescent image of nanorod bundles at 980 nm excitation wavelength and Figure S12 (see Supplementary Information) represents the corresponding optical image of nanorod bundles. The fluorescent image clearly demonstrates strong green emission throughout the bunch of Y_1.94_O_3_:Ho^3+^_0.02_/Yb^3+^_0.04_ nanorod bundles at different places. Further, PL mapping was performed at the same location from where the fluorescent image was taken (Fig. 2b) to explore the 2D spatial distribution of PL intensity on the surface of Y_1.94_O_3_:Ho^3+^_0.02_/Yb^3+^_0.04_ nanorod bundles. It is evident from Fig. 2b that the PL intensity distribution is not uniform. This is due to the fact that the topological surface of nanorod bundles are not uniform due to random selection of area and bunch of bundles being located at different heights. Furthermore, to explore a logistic behind observed non-uniform 2D PL intensity distribution of the surface of nanorod bundles, two different nanorod bundles with different profile heights are randomly selected. The PL mapping was performed and shown in Fig. 2c. The PL mapping result reveals that the intensity distribution is almost similar in two different nanorod bundles with different profile heights except the difference in PL intensity as shown in the insets of Fig. 2c (from A to B). In order to probe the precise 2D PL intensity distribution of the surface of isolated nanorod bundles another area of the sample where nanorod bundles are located individually either in horizontal or vertical position are selected. Figure 3a exhibits the optical image of isolated horizontal positioned single nanorod bundle. The inset of Fig. 3a represents fluorescent image of nanorod bundle showing strong green emission throughout the bundle (using excitation wavelength of 980 nm). Figure 3b exhibits the PL mapping image of isolated horizontal positioned single nanorod bundle. Figure 3c shows the PL intensity distribution along the length of nanorod bundle from one end to other end of the nanorod bundle, respectively (from A to B). The result reveals that the distribution is almost uniform (variation in PL intensity is ~0.1% in same order of magnitude) from position A to B in both the cases except the two broad peaks originating from the both edges of the bundle as shown in Fig. 3c. Usually, the PL intensity distribution appears uniform with naked eye in many cases but after investigation, did not show as expected, which can impact the optical display significantly and such issues can also be resolved through present probing method. Even <0.1% difference in same order of PL intensity can be also examine through this technique. Such observations are highly important in many cases such as flat penal display devices and optoelectronic devices. Similarly, the PL intensity distribution along the diameter of isolated bundles at three different selected positions; left, middle and right sides (marked from A to B) of isolated nanorod bundle are also investigated and result is shown in Fig. 3d–f. The result reveals the exact variation in PL intensity distributions at different places along the diameter of nanorod bundle. Furthermore, we also performed PL mapping of slanted and vertically positioned nanorod bundles and results are shown in Fig. 4a–f. The exact variations in PL intensity distribution have been shown in insets of Fig. 4a–f. However, the observed small variations in PL intensity distribution in nanorod bundles having different orientations are due to non-ideal alignment of nanorod in bundle shape. Hence, above PL mapping results justify its utility as a powerful tool to investigate the PL intensity distribution in luminescent materials.

Thus, in the light of above observations, we have found that the results of 2D spatially resolved PL distribution in nanorod bundles provide a new approach for acquiring the essential information about the PL intensity at different locations on the surface of nanostructures, which is highly desirable to investigate the uniform photo emission from flat panel optical display devices. In addition to this, PL mapping is a non-destructive essential tool for estimation of PL intensity distribution for various surface based luminescent nanophosphor applications such as security ink based bar codes, luminescent materials assisted solar concentrator for energy harvesting in photovoltaic and bio-labelling etc.

## Discussion

We have successfully demonstrated a strategy for the synthesis of highly-luminescent dual mode upconverting Y_1.94_O_3_:Ho^3+^_0.02_/Yb^3+^_0.04_ nanorod bundles by facile hydrothermal route. These highly luminescent nanorod bundles exhibit strong green emission centered at 549 nm upon 449 nm and 980 nm excitation wavelengths with quantum efficiencies ~6.3% and ~1.1%, respectively. The SEM results confirm that the bundles are composed nanorods. The TEM/HRTEM results exhibit that the individual nanorod are ~100 nm in diameter and ~1–3 μm in length. The possible growth mechanism for formation of nanorod bundle is proposed on the basis of observed experimental results. For the first time two dimensional spatially resolved photoluminescence intensity distribution in upconverting nanorod bundles using PL mapping microscopic technique has been studied. The present 2D spatial PL mapping intensity distribution results of nanorod bundles assure the proper substitutions of Ho^3+^(dopant), Yb^3+^(co-dopant) in Y_2_O_3_ (host lattice) nanorod bundles. Moreover, our finding could bring a better understanding about the PL intensity distribution in luminescent materials and provides a new direction that is extremely important for next generation photo emission based displays applications.

## Methods

### Materials

The precursors; Y_2_O_3_ (99.99%), Ho_2_O_3_ (99.99%), YbCl_3_:6H_2_O (99.99%), N-cetyl-N,N,N-trimethyl ammonium bromide (C_19_H_42_BrN, CTAB) and HNO_3_ (A.R. grade) were purchased from Sigma-Aldrich and used without further purification.

### Synthesis of Dual Mode Y_2_O_3_: Ho^3+^/Yb^3+^ Nanorod Bundles

The facile hydrothermal method was used for synthesis of dual mode Y_1.94_O_3_:Ho^3+^_0.02_/Yb^3+^_0.04_ nanorod bundles. The optimization activator and sensitizer of upconversion process in Y_2_O_3_ host lattice were optimized in our pervious report^15^. In present investigation, we have focused on the PL intensity distribution in nanorod bundle. In a typical synthesis, the stoichiometric amount of Y_2_O_3_ and Ho_2_O_3_ were dissolved in 20 ml D. I. water. Few drops of nitric acid were added to solution while stirring at 80 °C until the solution become transparent. Further, the precursors; YbCl_3_:6H_2_O and CTAB were dissolved in deionized water. All the above solutions were mixed by vigorous stirring at room temperature. Ethanol solution (1:5 ethanol: water) was added to the above mixed solution. Sodium hydroxide solution was added drop wise to the above solution till pH ~13 was achieved. Then, this solution was kept in hydrothermal vessel at 185 °C for 8 hours in box furnace. The pH is very crucial parameter for controlling the shape and size of nanorod bundles composed of individual dimensions of nanorod because these nanorod based bundles originate rapidly with closer nuclei formation as a co-nuclei effect at higher pH leads anisotropic growth during metal nitrate precursor to hydroxide formation. The obtained white precipitates was centrifuged several times with de-ionized water at 5000 rpm and then dried at 100 °C. The dried white powder was further sintered at 1000 °C for 5 hours to obtain the final product.

### Measurements and Characterizations

The crystal structure and composition of sample was identified by powder X-ray diffraction (XRD) using a Bruker AXS D8 Advance X-ray diffractometer, using Cu Ka_1_ radiation (λ = 1.5406 Å). The Raman spectrum was recorded using Renishaw inVia Raman spectrometer, with an excitation source of 514 nm. The thermogravimetric analysis (TGA) was carried out using a thermal analysis instrument with a heating rate of 10 °C min^−1^ in an air flow of 100 mL min^−1^. The surface morphology and energy dispersive X-ray analysis (EDAX) were examined by using field emission scanning electron microscope (FESEM) Carl ZEISS-SUPRA 40 VP equipped with EDAX facility. Transmission electron microscopy (TEM) and high-resolution transmission microscopy (HRTEM) were performed using a Tecnai G2 S-Twin with a field emission gun operating at 300 kV. The downconversion/downshift PL measurement was recorded using an Edinburg FLS900 fluorescence spectrometer equipped with 450 W xenon lamp as excitation source. The upconversion emission spectra were recorded using with an external power-controllable 980 nm diode laser (power density = 150 Wcm^−2^). To estimate the absolute luminescence quantum efficiency of nanorod bundles, an integrating sphere equipped with an Edinburgh spectrometer (Model FLS900) instrument has been used for measuring the integrated fraction of luminous flux and radiant flux with the standard method^1^. The time-resolved PL spectroscopy has been performed using Edinburg FLS900 fluorescence spectrometer where microsecond flash lamp acts as source of excitation. The PL mapping was carried out using WITec alpha 300 R + confocal PL microscope system where a diode laser of wavelength 980 nm act as source of excitation.

## Additional Information

**How to cite this article**: Kumar, P. *et al*. Experimental observation of spatially resolved photo-luminescence intensity distribution in dual mode upconverting nanorod bundles. *Sci. Rep.* **7**, 42515; doi: 10.1038/srep42515 (2017).

**Publisher's note:** Springer Nature remains neutral with regard to jurisdictional claims in published maps and institutional affiliations.

## Supplementary Material

Supplementary Information
