# Supplementary material for: Experimental observation of spatially resolved photo-luminescence intensity distribution in dual mode upconverting nanorod bundles
Source: Sci Rep. 2017 Feb 13;7:42515. doi: 10.1038/srep42515 (PMC5304174; doi:10.1038/srep42515)
Supplement: Supplementary Information [file srep42515-s1.doc]

**Supplementary Information**

**Experimental observation of spatially resolved photo-luminescence intensity distribution in dual mode upconverting nanorod bundles^†^**

Pawan Kumar**^1^**^,^**^2^**, Satbir Singh**^1^**^,^**^2^**, V. N. Singh**^3^**, Nidhi Singh**^4^,** R. K. Gupta**^5^** and

Bipin Kumar Gupta^1,*^

*^1^Luminescent Materials and Devices Group, Materials Physics and Engineering Division, CSIR- National Physical Laboratory, Dr K S Krishnan Road, New Delhi, 110012, India*

*^2^Academy of Scientific and Innovative Research (AcSIR), CSIR-National Physical Laboratory Campus , Dr K S Krishnan Road, New Delhi 110012, India*

*^3^Advanced Materials and Devices Group, Physics of Energy Harvesting Division, CSIR - National Physical Laboratory, Dr. K. S. Krishnan Road, New Delhi, 110012, India*

*^4^Metals, Alloys and Composites for Energy Applications Group, Physics of Energy Harvesting Division, CSIR - National Physical Laboratory, Dr. K. S. Krishnan Road, New Delhi, 110012, India*

***^5^****Department of Chemistry, Pittsburg State University, Pittsburg, KS, 66762, USA*

***E-mail:** [**bipinbhu@yahoo.com(B.K.G.)**](mailto:bipinbhu@yahoo.com(B.K.G.))

**
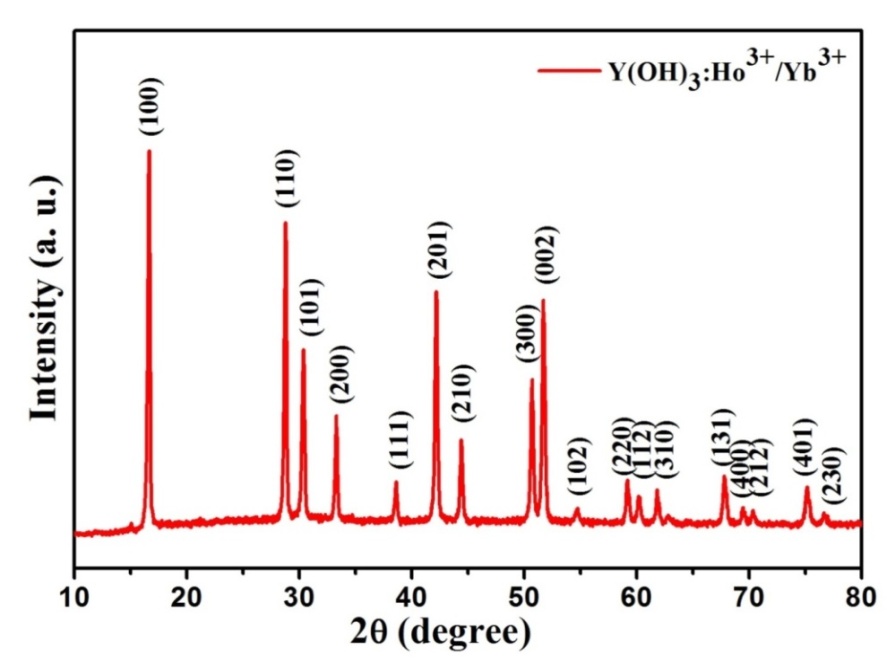
**

**Figure S1**. XRD pattern of Y_1.94_(OH)_3_: Ho^3+^_0.02_/Yb^3+^_0.04_ nanorod bundles.


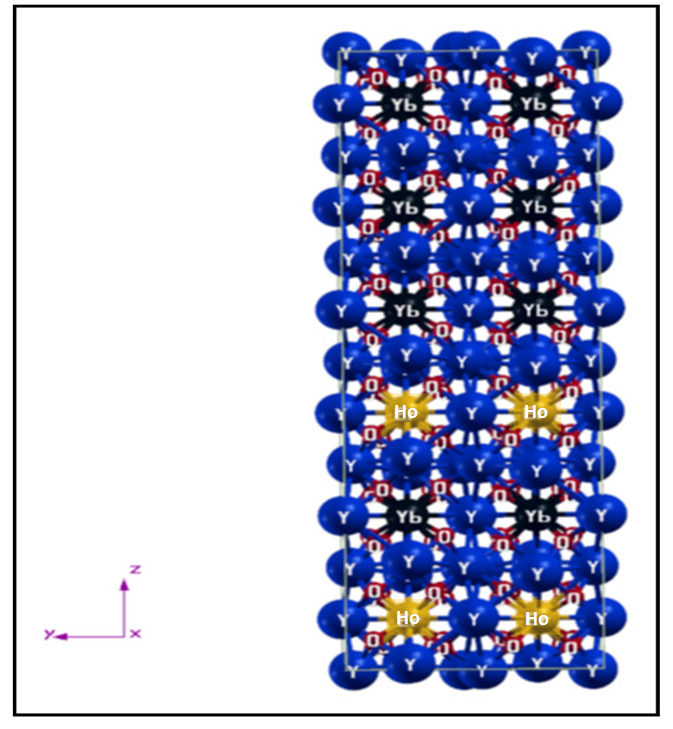


**Figure S2.** Proposed cubic crystal structure of Y_1.94_O_3_:Ho^3+^_0.02_/Yb^3+^_0.04_ nanorod bundles where Y atoms are replaced by Ho and Yb atoms.


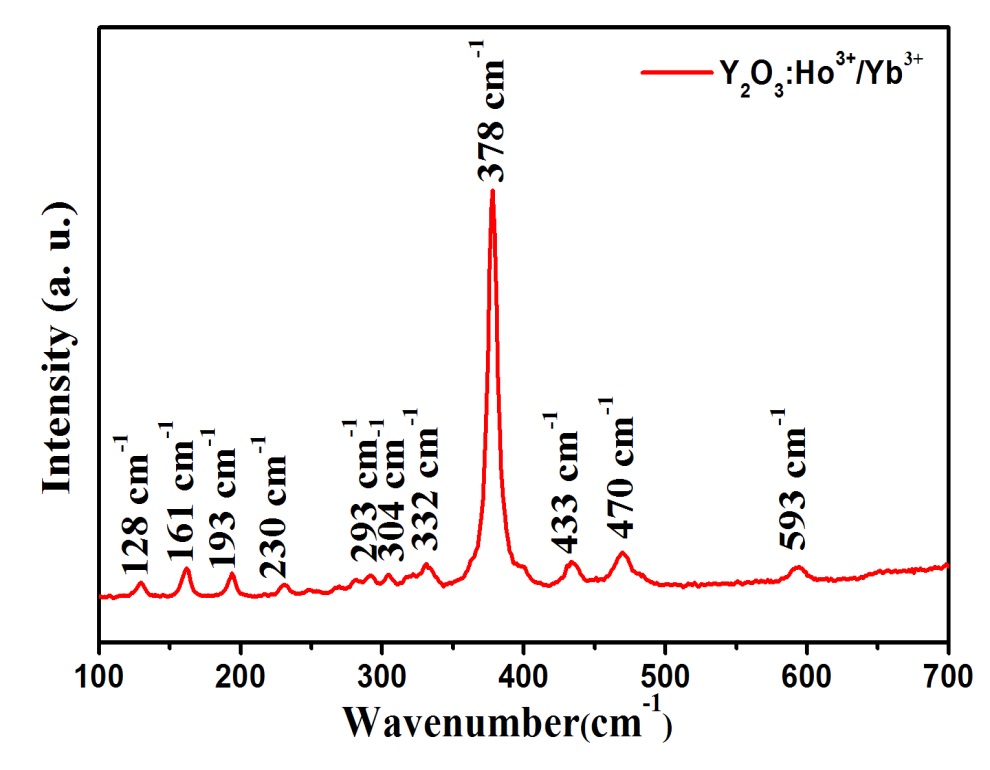


**Figure S3.** Raman spectrum of Y_1.94_O_3_: Ho^3+^_0.02_/Yb^3+^_0.04_ nanorod bundles.


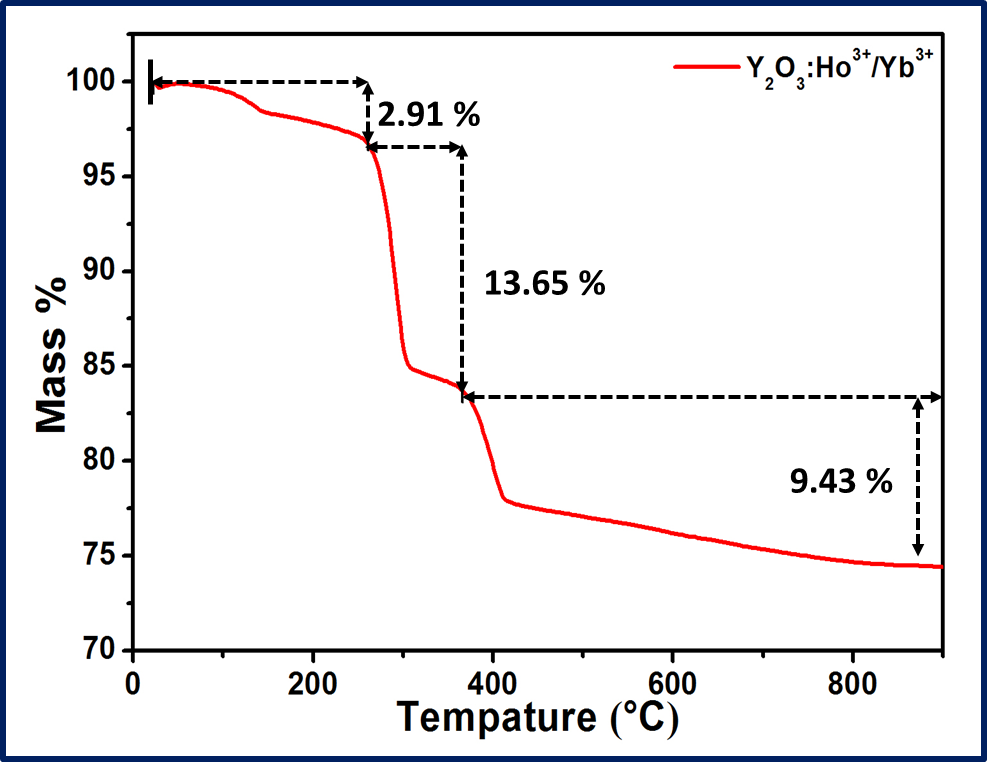


**Figure S4.** TGA graph of as-synthesized Y_1.94_(OH)_3_: Ho^3+^_0.02_/Yb^3+^_0.04_ nanorod bundles.


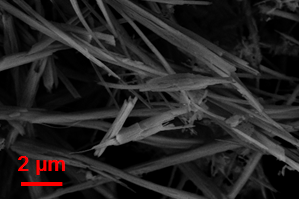


**Figure S5**. SEM image of Y_1.94_(OH)_3_: Ho^3+^_0.02_/Yb^3+^_0.04_ nanorod bundles.


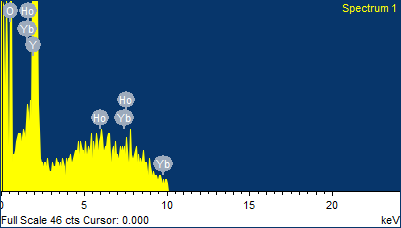


**Figure S6.** EDAX spectrum of Y_1.94_O_3_:Ho^3+^_0.02_/Yb^3+^_0.04_ nanorod bundles which show the presence of Y, O, Yb and Ho elements.


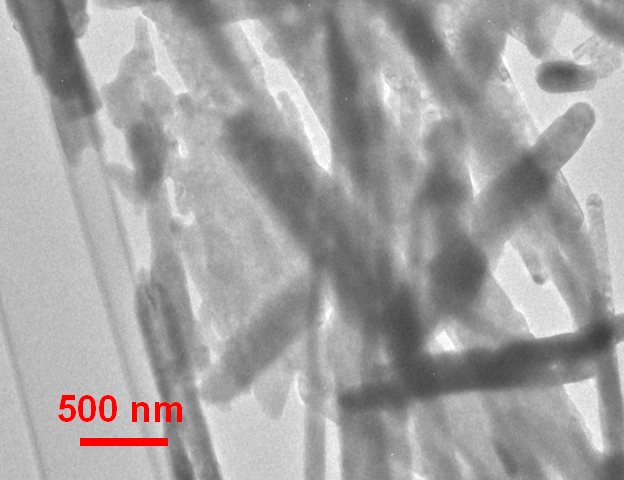


**Figure S7.** TEM image of nanorod bundle taken from different selected areas of Y_1.94_O_3_:Ho^3+^_0.02_/Yb^3+^_0.04_ nanorod bundles.


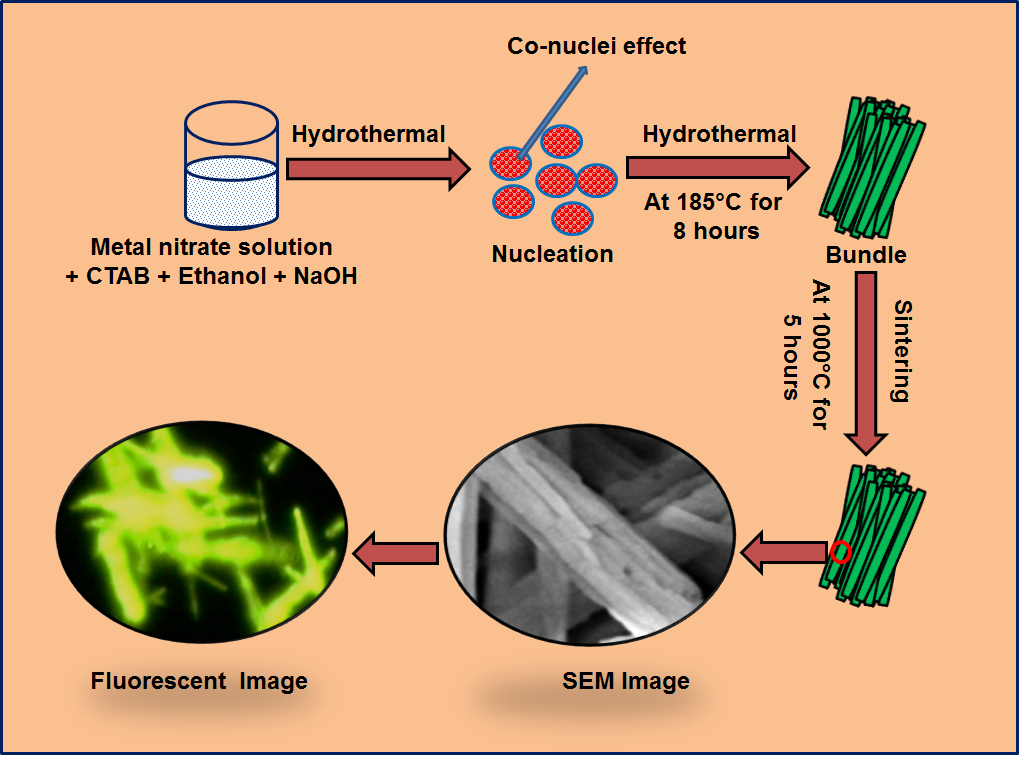


**Figure S8**. Proposed mechanism for the growth of nanorod bundles.

**
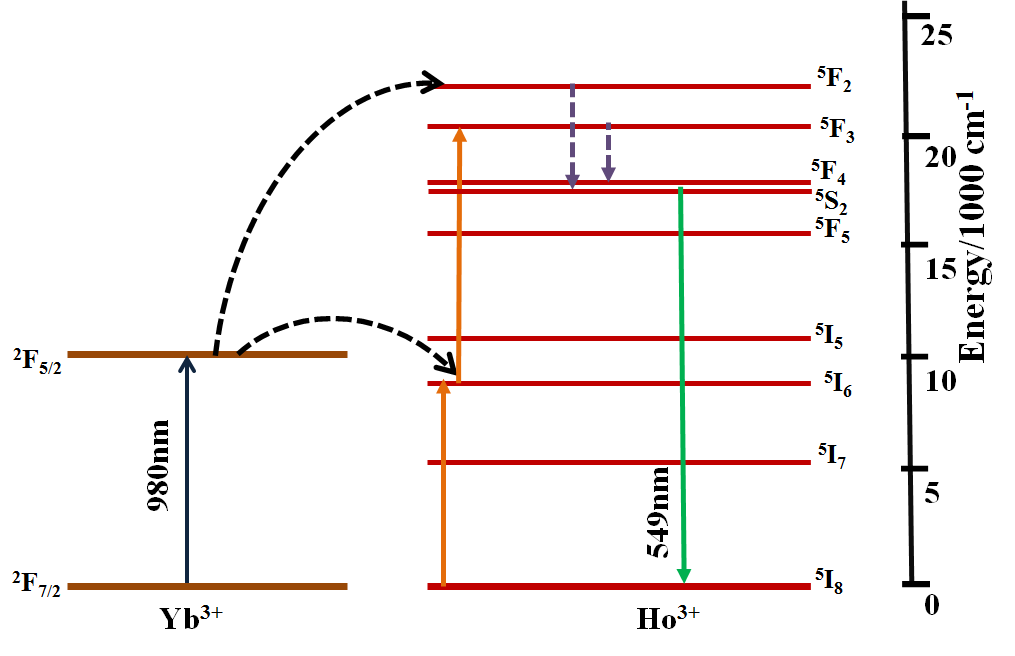
**

**Figure S9.** Proposed energy level diagram for upconversion process inY_1.94_O_3_:Ho^3+^_0.02_/Yb^3+^_0.04_ nanorod bundles.


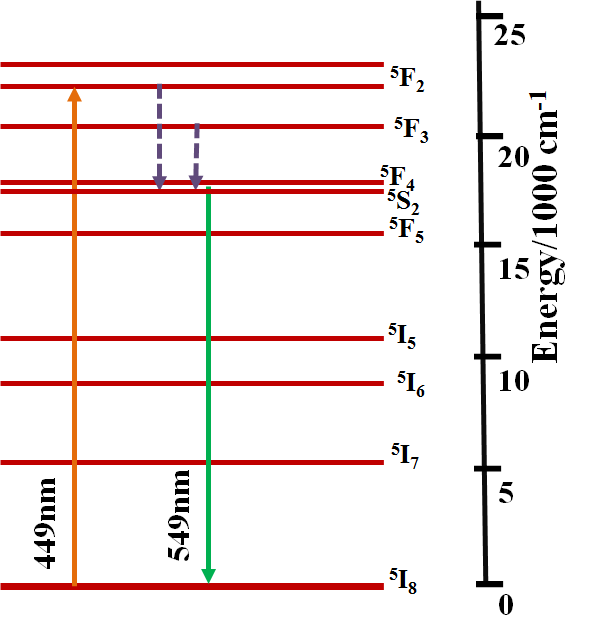


**Figure S10.** Proposed energy level diagram for down conversion process inY_1.94_O_3_:Ho^3+^_0.02_/Yb^3+^_0.04_ nanorod bundles.

In the confocal microscope, the laser light is conveyed via single-mode optical fiber. This type of fiber transmits only a single transversal mode (Gaussian beam), which can be focused to a diffraction limited spot. The light reflected by the sample is gathered by the same objective and is directed as a parallel beam toward the top of the microscope. Here, the light is focused onto a colour video camera or a multi-mode optical fiber. The core of this multi-mode optical fiber acts as a pinhole for confocal microscopy. The laser is raster-scanned across the sample by scanning the sample in all axes and the image is acquired line by line. Using fibers for beam delivery and signal pick-up is very convenient because bulky lasers and detectors can be placed far from the detecting microscope.^1,2^

The optimum pinhole diameter depends on the optical properties of the microscope objective along with the wavelength employed and can be calculated using the following formula:

**D ≤ λ · v · M/(N A · π)**

where λ is the wavelength of the laser, M is the magnification and NA is the numerical aperture of the microscope objective. The property v is given in optical coordinates and should be 2.5 for the best depth resolution and 0.5 for maximum lateral resolution.

**
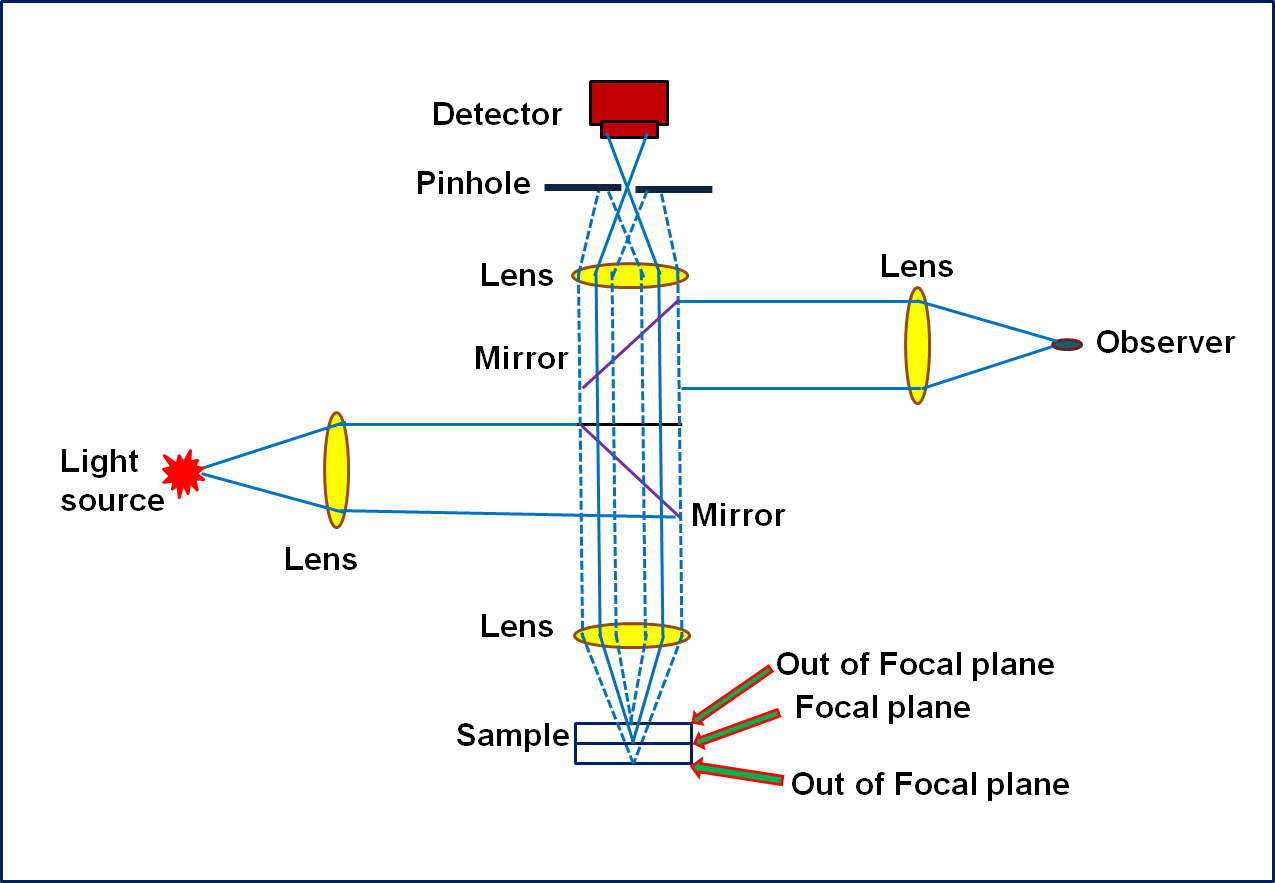
**

**Figure S11.**Schematic diagram of confocal microscope.

**
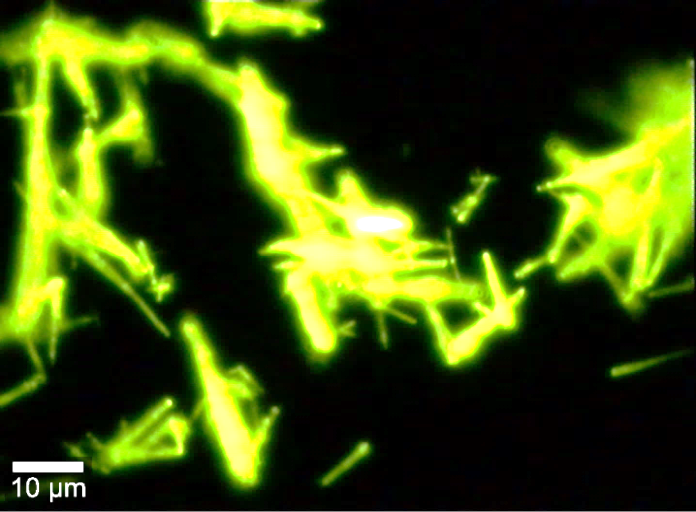
**

**Figure S12.** Optical image of Y_1.94_O_3_:Ho^3+^_0.02_/Yb^3+^_0.04_ nanorod bundles.

**References**

1. Pawley, J. B. Handbook of Biological Confocal Microscopy (3rd ed.). Berlin: Springer (2006).
2. <http://witec.de/>.
